# The influence of visual input and attention on gait initiation in people with Parkinson disease

**DOI:** 10.1371/journal.pone.0342661

**Published:** 2026-02-18

**Authors:** Chelsea Parker Duppen, Jenevieve Surkin, Shefaali Mahendar, Jordan Saunders, Jenna Cole, Nina Browner, Michael D. Lewek

**Affiliations:** 1 Human Movement Science Curriculum, University of North Carolina at Chapel Hill, Chapel Hill, North Carolina, United States of America; 2 Department of Physical Therapy, Virginia Commonwealth University, Richmond, Virginia, United States of America; 3 Joint Department of Biomedical Engineering, University of North Carolina at Chapel Hill, Chapel Hill, North Carolina, United States of America; 4 Department of Biostatistics, University of North Carolina at Chapel Hill, Chapel Hill, North Carolina, United States of America; 5 Department of Exercise and Sport Science, University of North Carolina at Chapel Hill, Chapel Hill, North Carolina, United States of America; 6 Department of Neurology, University of North Carolina at Chapel Hill, Chapel Hill, North Carolina, United States of America; 7 Division of Physical Therapy, Department of Health Sciences, University of North Carolina at Chapel Hill, Chapel Hill, North Carolina, United States of America; Iran University of Medical Sciences, IRAN, ISLAMIC REPUBLIC OF

## Abstract

**Introduction:**

Gait initiation relies on the integration of postural control, sensory input, and attention. All three components are impaired in Parkinson disease, which may contribute to characteristic gait initiation deficits, including shorter, slower first steps and smaller anticipatory postural adjustments. Understanding how sensory and attentional demands influence gait initiation could inform interventions that target underlying mechanisms rather than focusing solely on managing symptoms. This study examined the roles of vision and attention on gait initiation in people with Parkinson disease compared to older adult controls. We hypothesized that altering visual input and attentional demands would worsen gait initiation in both groups, with stronger effects in people with Parkinson disease. We also expected an interaction between visual input and attentional demands for people with Parkinson disease, further exacerbating impairment.

**Methods:**

Sixteen people with Parkinson disease (Hoehn & Yahr stages I-III, on medication), and 16 older adults (aged 55+) initiated gait under four visual conditions: unaltered input, partial occlusion, full occlusion, and additional visual stimuli, each performed with and without a cognitive dual task. We measured first step length, first step speed, and anticipatory postural adjustment size to compare between groups and conditions.

**Results:**

No interaction effects between group and condition were observed (all p ≥ 0.159). Full visual occlusion resulted in reduced first step length, first step speed, and anteroposterior anticipatory postural adjustment size (p ≤ 0.006). Partial occlusion resulted in decreased first step length and increased mediolateral anticipatory postural adjustment size (p ≤ 0.049). Gait initiation under a cognitive-motor dual task condition resulted in decrements across all variables (p ≤ 0.007).

**Conclusions:**

Reduced visual input and increased attentional demands impair gait initiation in older adults and people with Parkinson disease. These findings highlight the roles of visual input and attention during gait initiation, but suggest visuo-attentional deficits may not uniquely contribute to hypokinesia in this population.

## Introduction

Gait initiation is a complex motor task requiring postural control, sensory processing, and attention, all of which are compromised in Parkinson disease (PD) [1–3]. During gait initiation, people with Parkinson disease (PwP) demonstrate shorter first step lengths, slower first step speeds, and decreased anticipatory postural adjustment (APA) sizes [4,5]. Current pharmacological and rehabilitative interventions can temporarily improve these symptoms, but their benefits are limited and often short-lived [6–9], highlighting the importance of understanding the mechanisms underlying these impairments rather than focusing solely on symptom management. Despite this, the specific contributions of sensory processing and attention to hypokinesia and bradykinesia during gait initiation for PwP remain poorly understood.

Vision and visual processing play a critical role in the control of continuous walking [10–12]. In PwP, both excessive (i.e., overly stimulating) and insufficient (i.e., occluded) visual input during continuous walking can lead to shorter step lengths, slower gait speed, and episodes of freezing of gait (FOG) [13–17], reflecting a continued reliance on visual input despite impairments in vision and visual processing [18,19]. While the effects of visual input on continuous walking in PwP are well documented, gait initiation is a distinct task with less visual flow. This decrease in available visual information could either lessen reliance on vision or increase it, as participants may depend more heavily on stable visual cues to initiate movement safely. Thus, findings from continuous walking cannot be assumed to generalize to gait initiation.

Evidence from unimpaired older adults highlights the importance of visual input for gait initiation. When initiating gait with eyes closed, older adults exhibit smaller APAs, shorter first step lengths, and slower first step speeds, whereas younger adults are largely unaffected [20]. These findings suggest that older adults rely more heavily on vision for dynamic balance and gait initiation, a tendency also seen in PwP during balance tasks [21,22]. Although the specific role of visual input during gait initiation for PwP remains unclear, the fact that visual cues can enhance the initiation process [7,23] suggests that vision may contribute to the regulation of gait initiation movement patterns for PwP as well.

The role of attention in gait initiation is also important to consider because decreased attentional resources from a concurrent cognitive task (i.e., dual task) are known to negatively impact gait speed, arm swing, stride length, and foot strike angle in PwP during continuous walking [24,25]. Gait initiation is less automatic than continuous walking, and therefore requires greater cognitive and attentional resources [26]. Consequently, the attentional deficits seen at baseline in PwP may contribute to the hypokinetic and bradykinetic movements observed during gait initiation.

Despite this potential link, research examining the effects of reduced attentional resources on gait initiation in PwP remains limited. Although existing studies have reported declines in gait initiation performance during concurrent cognitive tasks for older adults and neurologic populations (including PwP), either participants were not instructed to prioritize the cognitive task [27–29], or the chosen attention-demanding task was ended prior to first step completion [30–33]. Thus, their results may not reflect real-world scenarios, where people often shift their focus to demanding cognitive activities (e.g., holding a conversation or planning a shopping list in their head) throughout the process of initiating gait, and consequently have less attentional resources to allocate to the task.

To complicate matters further, visual information cannot be used effectively without sufficient attentional resources. Visuo-cognition, which includes visuo-perception (recognizing stimuli), visuo-spatial processing (understanding spatial relationships), and visuo-construction (creating or manipulating visual representations), relies heavily on attention to interpret visual input and is therefore susceptible to dual-task scenarios. PwP, who demonstrate baseline impairments in visuo-cognition [34], also demonstrate impairments in pre-attentive processing (unconscious processing of visual stimuli) [19], requiring even greater attentional resources to interpret visual stimuli. This is particularly problematic because visuo-cognition is integral to safe ambulation and contributes to postural stability [34,35]. Impairments in visuo-cognition are thought to produce a “clamping” of postural sway during quiet stance, which may limit the weight shift needed to initiate gait [34,36]. Consequently, PwP, who already experience impairments in postural control [37], may show further declines in gait initiation when attentional resources are taxed by concurrent cognitive demands.

The purpose of this study was to investigate how visual input, sustained attentional demands, and their interaction influence hypokinesia and bradykinesia during gait initiation in PwP. We included an unimpaired older adult control group to help distinguish changes related to typical aging from those specific to PD. Because both older adults and PwP rely heavily on visual input to guide movement [16,20,34], we hypothesized that altering visual input, through occlusion or by adding extraneous stimuli, would reduce first step length, first step speed, and APA sizes in both groups, with PwP showing greater impairment. Similarly, because cognitive-motor dual tasking disrupts gait in both populations [24,25,38], we expected the addition of an attention-demanding cognitive task to further worsen gait initiation metrics in both groups, particularly for PwP. Finally, we predicted that combining partial visual occlusion, which increases demands on visuo-cognition by reducing available visual information, with an attention-demanding cognitive task would produce the largest impairments in PwP due to their baseline visuo-cognitive deficits and dual-task vulnerability [24,34].

## Methods

### Participants

We recruited adults aged 55 years or older with or without a diagnosis of PD. Participants were included if they were able to ambulate without assistance or an assistive device and had normal or corrected-to-normal vision (Snellen eye chart score better than 20/30 using typical eyewear). Exclusion criteria included Parkinson-related or unrelated dementia (Montreal Cognitive Assessment score < 23) [39], orthopedic impairments impacting gait, or any neurologic condition other than PD. Participants with PD were eligible if they had a confirmed diagnosis of idiopathic PD and were within Hoehn & Yahr stages I-III. Testing for participants with PD was conducted during their ‘on’ medication state. All participants signed a written informed consent form approved by the University of North Carolina at Chapel Hill Institutional Review Board (IRB # 23–3022). Recruitment began on April 1^st^, 2024 and concluded on November 15^th^ 2024.

### Overall design

Prior to testing, all participants completed the Mini-BESTest, and participants with PD also completed the Freezing of Gait Questionnaire (FOGQ). Testing consisted of a single session in which participants initiated gait under four visual conditions: 1) no visual occlusion, 2) partial visual occlusion, 3) full visual occlusion, and 4) added visual stimuli. Each condition was performed both with and without a secondary attention-demanding cognitive task (i.e., cognitive-motor dual task). The ‘no visual occlusion’ condition was always completed first as a baseline, while the order of the remaining visual conditions was randomized. Within both groups (older adults and PwP), participants were counterbalanced to begin each visual condition either with or without the dual task. Six trials were completed for each condition, based on prior work indicating 3–5 trials were necessary to get an accurate measurement of APA size and first step length [40], and our desire to maintain this accuracy in the event of data loss. Participants were not initially instructed which foot to use for the first step. After three trials were initiated with the same limb, they were verbally cued to begin any remaining trials with the contralateral limb to ensure that each condition included three trials initiated with each foot.

All gait initiation trials were completed overground on a 6.1m (20 ft) Zeno instrumented walkway (Zeno, Protokinetics, Havertown, PA). Tape was placed on the mat to indicate starting foot position on the mat. Participants began gait initiation trials standing on the mat to allow for an analysis of center of pressure (COP) shifts, first step length, and first step speed. A brief still period was recorded prior to each gait initiation trial, followed by the researcher cueing participants to begin walking by saying “whenever you’re ready” in a flat tone. This method was used instead of saying “go” to reduce the influence of an auditory cue on gait initiation and facilitate more volitional initiation of gait [6,41]. Participants walked across the sensor mat, returned to the start, and repeated the process for subsequent trials. Participants wore a gait belt throughout testing that did not restrict movement. Participants were not allowed assistance or an assistive device to complete gait initiation trials.

### Dual task

The attention-demanding cognitive task was a forward digit span test, chosen for its adaptability to individual working memory capacity [42], and its lack of rhythmic or visual cues. Before data collection, participants completed the task while seated to establish the threshold at which accuracy dropped below 100%. This threshold determined the number of digits used during subsequent dual-task trials. Participants received a unique set of numbers before beginning each trial, as determined by a random number generator, and were instructed to recall them after completing the trial to avoid complications of speech motor impairment or pacing of steps [43]. To encourage participants to prioritize the cognitive task during gait initiation trials, they were told that the number of incorrect responses would be recorded.

### Partial visual occlusion

The inferior portion of goggles was blocked to prevent participants from viewing their feet. To standardize partial visual occlusion, a target was placed on a wall 5’ from the participant at 90% of their height. As opposed to cues for upright posturing, researchers instructed participants to stand comfortable to account for any increased neck or trunk flexion PwP may demonstrate. Vision was obstructed on the goggles such that the participants were able to view the target at 90% of their height, while markings situated 6” and 8” below the main target were not visible. Participants were not cued on where to direct their gaze during gait initiation trials, but were encouraged to avoid excessive forward trunk flexion in an attempt to view the floor while walking.

### Full visual occlusion

Participants wore blacked-out goggles to eliminate visual input. They were closely guarded for safety, but no physical assistance was provided unless necessary. Trials requiring assistance during the first step were excluded from analysis. If a participant could not complete any unassisted trials, that condition was omitted. This occurred for one participant in the PwP group, who was unable to complete either full visual occlusion condition (with or without a dual task).

### Added visual stimuli

Six-foot barriers, arranged to form a 4-ft-wide corridor along the 20-ft sensor mat, were used to introduce visuospatial constraints, as prior studies show PwP take shorter steps in narrow spaces [16]. Additional visual distractors were mounted on the barriers to simulate a cluttered environment.

### Data management and analysis

First step length and first step speed during gait initiation were extracted directly from Protokinetics software and were normalized to participant height. Mediolateral (ML) and anteroposterior (AP) APA sizes were measured as the maximal COP excursions laterally toward the initial stepping limb and posteriorly, respectively, from the ‘still’ period until toe-off of the stepping extremity. For each variable of interest, trials were averaged within each condition for each participant.

Data were analyzed using SPSS (v28; IBM Corp, Armonk, NY). To determine the effects of visual condition (no visual occlusion, partial visual occlusion, full visual occlusion, and additional visual stimuli), attentional condition (dual task or no dual task), and group (older adults or PwP) on the gait initiation outcome measures, we employed linear mixed effect models for first step length, first step speed, and ML and AP APA sizes. The fixed effects were visual condition, attentional condition, and group, with visual condition and attentional condition as repeated measures. Participants were included as a random effect to account for potential individual variability in response to the different conditions. For all models, we implemented an unstructured covariance structure.

Given the complexity of our model and the limited sample size, we could not include sequence (order of visual conditions) or order (dual task vs. no dual task first) in the analysis. Visual conditions were randomized and attentional conditions counterbalanced, so any variance due to sequence or order is effectively random.

When significant main effects or interactions were identified, planned post-hoc analyses were conducted in the form of pairwise comparisons using estimated marginal means (EMMs). Bonferroni corrections were applied to account for multiple comparisons.

The normality of our outcome measures was evaluated using the Shapiro-Wilk test. First step speed demonstrated acceptable normality (p > 0.05). However, descriptive analysis revealed a small, but statistically significant, negative skewness for first step length (skewness = − 0.490, p < 0.001). This non-normality was best addressed via a square transformation (skewness = 0.148, p = 0.071). Mediolateral and AP APA sizes also exhibited non-normality (both p < 0.001). The non-normality of ML APA size was minimal, and thus could be managed by the robustness of Linear Mixed Effects Models (LMMs) without transformation (skewness = 0.239, p < 0.001) [44]. Anteroposterior APA sizes were effectively transformed using modified logarithmic transformation (log(AP APA + 1)), significantly reducing skewness to an acceptable level (skewness = 0.482, p < 0.001) for LMM analysis.

An a priori power analysis was completed based on estimates from prior within-subjects comparisons of experimentally altered first step length [7]. Using G*Power (3.1.9.6) with an estimated effect size of *f* = 0.25, α = 0.05, power = 0.90, correlation = 0.6, and ε = 0.6, the minimum sample size was 24 participants (12 per group). We recruited 16 participants per group to accommodate potential data loss.

In addition to LMMs for each gait initiation outcome variable of interest, we conducted exploratory Spearman rank correlation tests to assess the relationship between APA size and first step length and speed at baseline as well as the relationship between change in APA size and change in first step length and speed across all other trials. These analyses are based on prior work questioning the role of APA size on first step performance [45]. Change scores were based on baseline gait initiation metrics.

## Results

Sixteen PwP and 16 unimpaired older adults participated (Table 1). One participant from the PwP group was unable to complete both full visual occlusion conditions (dual task or no dual task). Due to the nature of our LMM, we were able to use their data for the rest of the analyses.

**Table 1:** Participant Characteristics (mean ± SD).

	PwP	Controls	p-value
**Age (years)**	72 ± 7	69 ± 7	0.206
**Sex**	7M, 9F	4M, 12F	0.264
**Comfortable Overground Gait Speed (m/s)**	1.08 ± 0.21	1.22 ± 0.18	**0.037**
**Overground Average Step Length (m)**	0.607 ± 0.100	0.676 ± 0.094	**0.036**
**Mini-BESTest Score**	21 ± 4	26 ± 2	**< 0.001**
**MOCA Score**	26 ± 2	28 ± 1	**0.001**
**Hoehn & Yahr Stage**	3 Stage 3, 6 Stage 2, 3 Stage 1	N/A	N/A
**UPDRS total score**	21 ± 13	N/A	N/A

*Note.* UPRDS and Hoehn & Yahr stages were not collected for 4 of the 16 PwP. Statistical tests used gait speed and step length values that were normalized to participant height, while the chart displays original, non-normalized values.

### First step length

**Fig 1:**
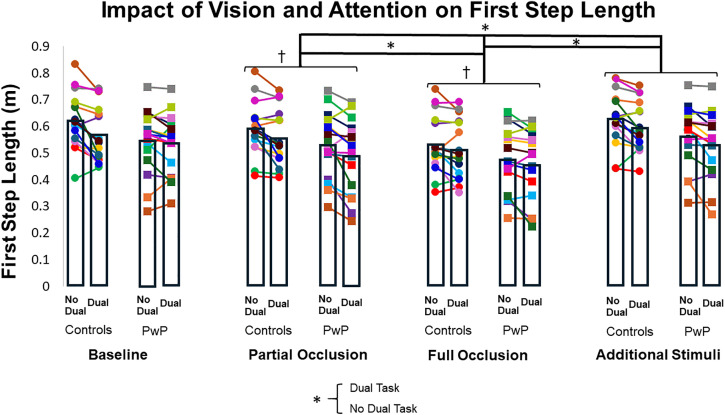
Impact of vision and attention on first step length. Each color circle represents a control participant’s average for each condition, and each color square represents a participant with PD’s average. Bar graphs represent the average for first step length for each group. † = different from baseline; * = difference between conditions.

There was no interaction effect between group and visual condition or group and attentional condition for first step length (F_(3, 30.039)_ = 0.301, ηp² = 0.029, p = 0.825; F_(1, 30.035)_ = 1.220, ηp² = 0.039, p = 0.278), indicating that both groups exhibited a similar pattern of changes across conditions. Additionally, there was no significant main effect of Group (F_(1, 29.967)_ = 2.815, ηp² = 0.086, p = 0.104). However, we found significant main effects of visual condition (F_(3, 30.039)_ = 32.287, ηp² = 0.763, p < 0.001) and attentional condition (F_(1, 30.035)_ = 20.912, ηp² = 0.410, p < 0.001) (Fig 1 and Table 2). Specifically, first step length was shorter during the full visual occlusion condition compared to the baseline (least squares mean [LSM] difference ± standard error [SE] = −0.029 ± 0.003, 95% Confidence Interval (CI) [−0.038, −0.021], p < 0.001), partial occlusion (LSM = −0.020 ± 0.003, CI [−0.028, −0.012], p < 0.001), and additional visual stimuli conditions (LSM difference = −0.034 ± 0.004, CI [−0.045, −0.023], p < 0.001). Partial visual occlusion also resulted in shorter first step lengths compared to baseline (LSM difference = 0.009 ± 0.003, CI [−0.017, −0.001], p = 0.016) and additional visual stimuli conditions (LSM difference = 0.014 ± 0.003, CI [−0.023, −0.006], p < 0.001), but there was no difference between the baseline and additional visual stimuli conditions (LSM difference = −0.005 ± 0.002, CI [−0.011, 0.001], p = 0.216). The main effect of attentional condition indicated that adding a dual task resulted in reduced first step length (LSM difference = −0.012 ± 0.003, CI [−0.018, −0.007], p < 0.001).

**Table 2:** First step length averages across dual task conditions (m) (mean ± SD).

		Visual Conditions			
		Baseline	Partial Occlusion	Full Occlusion	Additional Stimuli
**No Dual Task**	**PwP**	0.546 ± 0.117	0.530 ± 0.119	0.476 ± 0.121	0.564 ± 0.113
	**Controls**	0.621 ± 0.102	0.591 ± 0.098	0.532 ± 0.105	0.627 ± 0.098
**Dual Task**	**PwP**	0.536 ± 0.110	0.487 ± 0.135	0.455 ± 0.130	0.530 ± 0.121
	**Controls**	0.569 ± 0.102	0.555 ± 0.102	0.511 ± 0.108	0.597 ± 0.090

*Note.* Statistical tests used first step length values that were normalized to participant height, while the chart displays original, non-normalized values. Comparisons between variables are described in the text.

### First step speed

**Fig 2:**
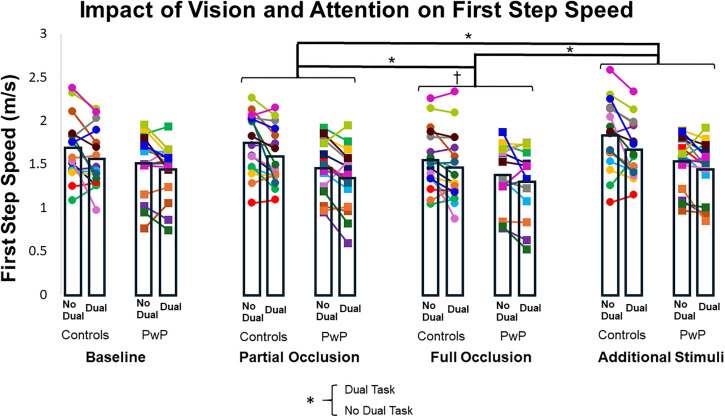
Impact of vision and attention on first step speed. Each color circle represents a control participant’s average for each condition, and each color square represents a participant with PD’s average. Bar graphs represent the average for first step speed for each group. † = different from baseline; * = difference between conditions.

There was no interaction effect between group and visual condition or group and attentional condition for first step speed (F_(3, 29.834)_ = 1.852, ηp² = 0.157, p = 0.159; F_(1, 29.946)_ = 0.819, ηp² = 0.027, p = 0.373), indicating that both groups exhibited a similar pattern of changes across conditions. There were, however, significant main effects of visual condition (F_(3, 29.834)_ = 16.179, ηp² = 0.619, p < 0.001) and attentional condition (F_(1, 29.946)_ = 18.237, ηp² = 0.379, p = 0.001) (Fig 2 and Table 3). First step speed was slower during full visual occlusion compared to baseline (LSM difference = −0.087 ± 0.018, CI [−0.137, −0.037], p < 0.001), partial visual occlusion (LSM difference = −0.080 ± 0.022, CI [−0.142, −0.018], p = 0.006), and additional visual stimuli (LSM difference = −0.128 ± 0.021, CI [−0.188, −0.068], p < 0.001) conditions. First step speed was also slower during partial visual occlusion than with additional visual stimuli (LSM difference = −0.048 ± 0.012, CI [−0.083, −0.014], p = 0.003). The main effect of attentional condition indicated that adding a dual task reduced first step speed (LSM difference = −0.067 ± 0.016, CI [−0.099, −0.035], p < 0.001). There was also a main effect of group (F_(1, 29.950)_ = 4.270, ηp² = 0.125, p = 0.048), such that older adults demonstrated faster first step speed compared to PwP (LSM difference = 0.133 ± 0.064, CI [0.002, 0.265], p = 0.048).

**Table 3:** First step speed averages across dual task conditions (m/s) (mean ± SD).

		Visual Conditions			
		Baseline	Partial Occlusion	Full Occlusion	Additional Stimuli
**No Dual Task**	**PwP**	1.504 ± 0.346	1.458 ± 0.309	1.373 ± 0.347	1.533 ± 0.293
	**Controls**	1.685 ± 0.350	1.739 ± 0.339	1.553 ± 0.347	1.816 ± 0.385
**Dual Task**	**PwP**	1.436 ± 0.304	1.340 ± 0.347	1.297 ± 0.377	1.434 ± 0.323
	**Controls**	1.556 ± 0.332	1.582 ± 0.315	1.460 ± 0.387	1.668 ± 0.326

*Note.* Statistical tests used first step speed values that were normalized to participant height, while the chart displays original, non-normalized values. Comparisons between variables are described in the text.

### ML and AP APA sizes

There was no interaction effect between group and visual condition or group and attentional condition for ML APA size (F_(3, 29.954)_ = 0.692, ηp² = 0.065, p = 0.564; F_(1, 29.529)_ = 0.043, ηp² = 0.001, p = 0.837), indicating that both groups exhibited a similar pattern of changes across conditions. Additionally, there was no significant main effect of Group (F_(1, 29.952)_ = 2.186, ηp² = 0.068, p = 0.150). There was a main effect of visual condition (F_(3, 29.954)_ = 2.999, ηp² = 0.231, p = 0.046) and attentional condition (F_(1, 29.528)_ = 8.573, ηp² = 0.225, p = 0.007) on ML APA size (Table 4). Specifically, ML APA size was larger during partial visual occlusion than at baseline (LSM difference = 0.003 ± 0.001, CI [5.882 x 10 ⁻ ⁶, 0.006], p = 0.049), with no differences between other visual conditions (all p ≥ 0.217). For attentional conditions, ML APA size was larger during the no dual task conditions compared to the dual task conditions (LSM difference = 0.003 ± 0.001, CI [0.001, 0.005], p = 0.007).

**Table 4:** ML APA averages across dual task conditions (m) (mean ± SD).

		Visual Conditions			
		Baseline	Partial Occlusion	Full Occlusion	Additional Stimuli
**No Dual Task**	**PwP**	0.016 ± 0.009	0.019 ± 0.011	0.016 ± 0.011	0.020 ± 0.010
	**Controls**	0.020 ± 0.009	0.026 ± 0.010	0.020 ± 0.010	0.022 ± 0.012
**Dual Task**	**PwP**	0.013 ± 0.008	0.015 ± 0.009	0.017 ± 0.010	0.013 ± 0.008
	**Controls**	0.018 ± 0.013	0.020 ± 0.013	0.018 ± 0.010	0.022 ± 0.012

*Note.* Comparisons between variables are described in the text.

Similar to findings for ML APA sizes, there were no interaction effects between group and visual condition or group and attentional condition for AP APA size (F_(3, 30.019)_ = 0.266, ηp² = 0.026, p = 0.849; F_(1, 30.011)_ = 0.583, ηp² = 0.019, p = 0.451) (Table 5), indicating that both groups exhibited a similar pattern of changes across conditions. Additionally, there was no main effect of group (F_(1, 29.979)_ = 3.923, ηp² = 0.116, p = 0.057). However, there were main effects of visual condition (F_(3, 30.044)_ = 15.919, ηp² = 0.614, p < 0.001) and attentional condition (F_(1, 30.010)_ = 31.637, ηp² = 0.513, p < 0.001). Specifically, full visual occlusion resulted in smaller AP APAs than baseline (LSM difference = −0.003 ± 0.001, CI [−0.005, −0.002], p < 0.001), partial visual occlusion (LSM difference = −0.002 ± 0.001, CI [−0.004, −0.001], p < 0.001), and additional visual stimuli conditions (LSM difference = −0.004 ± 0.001, CI [−0.006, −0.002], p < 0.001), with no other between-conditions differences (all p ≥ 0.059). The addition of a dual task also reduced AP APA size compared to the no dual task conditions (LSM difference = −0.004 ± 0.001, CI [−0.005, −0.002], p < 0.001).

**Table 5:** AP APA averages across visual and dual task conditions (m) (mean ± SD).

		Visual Conditions			
		Baseline	Partial Occlusion	Full Occlusion	Additional Stimuli
**No Dual Task**	**PwP**	0.029 ± 0.016	0.025 ± 0.017	0.021 ± 0.015	0.030 ± 0.018
	**Controls**	0.038 ± 0.017	0.040 ± 0.016	0.031 ± 0.016	0.040 ± 0.015
**Dual Task**	**PwP**	0.021 ± 0.014	0.019 ± 0.012	0.014 ± 0.009	0.022 ± 0.014
	**Controls**	0.031 ± 0.015	0.027 ± 0.015	0.023 ± 0.016	0.031 ± 0.016

*Note.* Comparisons between variables are described in the text.

### Relationship between APAs and first step performance

Spearman rank correlations revealed a significant positive relationship between first step length and ML APA size (ρ = 0.602, p < 0.001), first step length and AP APA size (ρ = 0.666, p < 0.001), first step speed and ML APA size (ρ = 0.419, p = 0.017), and first step speed and AP APA size (ρ = 0.384, p = 0.030) for the baseline trials.

Spearman rank correlations for change scores revealed a positive, albeit weak, relationship between change in ML APA size and change in first step length (ρ = 0.297, p < 0.001), change in AP APA size and change in first step length (ρ = 0.309, p < 0.001), change in ML APA size and change in first step speed (ρ = 0.160, p = 0.017), and change in AP APA size and first step speed (ρ = 0.341, p < 0.001).

## Discussion

Our hypothesis that adding an attention-demanding cognitive task during gait initiation would reduce first step length, first step speed, and APA sizes in PwP and unimpaired older adults was supported by the data. In contrast, our hypothesis that altering visual input by either occluding or adding visual stimuli would lead to performance decrements was only partially supported. Specifically, the additional visual stimuli condition did not negatively impact gait initiation metrics for either group. Additionally, our data did not support the hypothesis that PwP would experience greater challenges from changes in visual stimuli and attentional demands compared to older adult controls. Instead, PwP exhibited responses comparable to older adults across visual and attentional conditions. Lastly, there was no interaction between visual input and attentional demand for either group, contrary to our final hypothesis that PwP would demonstrate the most difficulty during partial visual occlusion with a dual task due to the high levels of visuo-cognition required. These findings suggest that vision and attention can influence gait initiation for both groups, but may not fully explain the presence of hypokinesia and bradykinesia observed during gait initiation in PwP.

Based on prior research suggesting that PwP experience greater difficulty with dual tasks and altered visual input than similarly-aged peers [16,46], it was surprising that we found no statistically significant group differences across visual and attentional conditions. Several factors may account for this. It is possible that there is genuinely no difference in how PwP and older adults respond to these conditions. Alternatively, our PwP cohort may have been too high-functioning to reveal subtle impairments, as most participants were classified as Hoehn & Yahr stages 1 or 2, indicating mild disease progression with minimal gait and postural control deficits. Only three participants were in stage 3, reflecting more moderate impairment. Finally, the lack of differences may reflect testing while PwP were ‘on’ medication. Although dopaminergic efficacy fluctuates throughout the day and declines as the disease progresses [8], our participants were tested when they reported optimal medication effectiveness. Future studies should examine PwP during ‘off’ medication periods and in later disease stages to fully determine whether group differences exist, and if these differences are mediated by dopaminergic medications.

The dual-task condition in our study reduced performance across all gait initiation metrics in both groups. This suggests that the cognitive task we employed may have imposed a higher cognitive load than tasks used in previous studies [27,28], which found no significant effects of cognitive-motor dual tasks on gait initiation in younger adults, older adults, or PwP. The digit span task was selected for its adaptability to each participant’s working memory capacity, which may explain why our results differ from studies using a generic n-back task that could have been easier for some participants and more difficult for others. Furthermore, participants were asked to recall the number sequence only after completing the trial, rather than during walking, requiring them to retain the sequence for an extended period (~20 seconds). This likely increased the cognitive and attentional demands, contributing to the larger dual-task costs observed. In addition, participants were encouraged to prioritize the cognitive task, whereas previous work asked participants to divide attention equally between cognitive and motor tasks [28]. Together, these findings underscore the importance of both task difficulty and prioritization strategies when assessing dual-task effects on complex motor behaviors such as gait initiation.

While cognitive load clearly influenced gait initiation, manipulations of visual input had a different pattern of effects. The additional visual stimuli condition did not alter first step length, first step speed, or APA sizes. This may be due to the choice of visual stimuli, which, though designed to mimic a visually distracting hallway, lacked the spatial constraints (e.g., narrowness or ceiling height) that previous studies have shown to impair gait in PwP [16,47]. It is possible that these performance decrements are driven more by spatial anxiety than by visual distraction alone. This supposition is consistent with earlier work suggesting that temporal and spatial constraints, rather than visual stimuli, may trigger gait impairments in PwP [48].

Another potential explanation is the static nature of the visual stimuli used in our study. Unlike dynamic environments, such as crowded grocery stores with moving people and carts, our stimuli may not have been complex enough to disrupt gait initiation. Additionally, reduced visual flow during gait initiation, compared to continuous walking, may account for the observed results. Because participants are relatively stationary during the preparatory phase, they process less dynamic visual information, which could limit the perceived complexity of the environment. In contrast, continuous walking exposes individuals to greater visual flow, even in static surroundings, potentially placing higher demands on the visual and attentional systems.

The partial visual occlusion condition yielded unexpected, but interesting results. As expected, first step length, first step speed, and AP APA size decreased compared to baseline. However, there was an unexpected increase in ML APA size, consistent across both groups. Previous research has shown that unimpaired populations often perform worse on postural control tasks when focusing on an internal locus of attention compared to an external one, such as a visual or cognitive task [49]. The prevailing theory is that directing attention to highly automatized processes like postural control can paradoxically impair their effectiveness. Although we did not explicitly cue participants to focus on mediolateral postural stability during gait initiation trials, verbal cues during equipment calibration may have influenced these outcomes. Specifically, participants were instructed to “stand as still as you can,” prior to each trial, which could have shifted their attention inward, inadvertently affecting performance during the preparatory phase of gait initiation (i.e., APA size and weight shift). Alternatively, when vision was partially occluded, this internal focus may have been disrupted, requiring attention to shift away from the static standing cue towards higher-level visuo-cognitive processes. Such a shift could have facilitated greater ML APA amplitudes, even exceeding baseline performance, which is consistent with the functional role of ML APAs (vs. AP APAs) in maintaining dynamic stability [50,51].

Although postural control is largely automatic, the weight shift preceding gait initiation likely requires motor preparatory attention to integrate sensory inputs and generate appropriate motor commands [52]. This perspective may help explain why ML APA size increased under partial visual occlusion yet decreased during the more attentionally demanding dual-task condition. One possibility is that both excessive and insufficient attentional engagement can disrupt the control of these preparatory adjustments. Another possibility is that the type of attention matters more than the amount. Directing attention toward external environmental or sensory cues may support balance and postural control, while dividing attention or shifting it inward may be detrimental. Further research is needed to clarify this relationship and determine the optimal level of attentional engagement, as well as the most beneficial focus of attention.

The increase in ML APA size during partial visual occlusion, despite reductions in first step length and speed, suggests that changes in APA size do not always translate directly to changes in first step performance. At baseline, first step length and speed were moderately to strongly correlated with both ML and AP APA sizes (ρ = 0.384–0.666, all p ≤ 0.030), indicating a clear relationship under typical conditions. However, correlations between change scores induced by altered visual and attentional demands were weaker (ρ = 0.160–0.341, all p ≤ 0.017), suggesting that the magnitude of change in APA size is not consistently mirrored by a proportional change in step length or speed. This pattern reinforces the idea that APAs primarily serve to maintain postural stability, and under challenging conditions, participants may prioritize stability over step execution. Our findings align with recent research indicating that postural control mechanisms and first step performance are related, but that the mere presence of a weak relationship does not suggest that changes in one will necessarily result in equivalent changes in the other [45,53]. Future research may benefit from considering gait initiation as comprising of two independent processes: one governing postural control and the other governing step execution.

Overall, visual and attentional manipulations led to similar performance decrements in both groups, suggesting these changes are not unique to PwP with mild to moderate impairment in the ‘on’ medication state. While this study provides important insights into the role of vision and attention during gait initiation, there are several noted limitations. First, the complex design and small sample size limited our ability to statistically control for the sequence of conditions. Although randomization and counter-balancing were used to minimize these effects, future studies with larger samples should account for potential practice or carryover effects more rigorously. Additionally, we used APA amplitude as an index of postural control, yet prior research suggests that APA duration may be more closely related to postural instability [54]. Future work should investigate how visual and attentional demands affect APA timing and duration to better understand their influence on the preparatory phase of gait initiation.
